# The nuclei of human adult stem cells can move within the cell and generate cellular protrusions to contact other cells

**DOI:** 10.1186/s13287-024-03638-y

**Published:** 2024-02-07

**Authors:** Carlos Bueno, David García-Bernal, Salvador Martínez, Miguel Blanquer, José M. Moraleda

**Affiliations:** 1 Medicine Department and Hematopoietic Transplant and Cellular Therapy Unit, Faculty of Medicine, Institute of Biomedical Research (IMIB), University of Murcia, 30120 Murcia, Spain; 2 Biochemistry, Molecular Biology and Immunology Department, Faculty of Medicine, University of Murcia, 30100 Murcia, Spain; 3 Instituto de Neurociencias de Alicante (UMH-CSIC), Universidad Miguel Hernandez, 03550 San Juan, Alicante, Spain; 4 Center of Biomedical Network Research on Mental Health (CIBERSAM), ISCIII, 28029 Madrid, Spain; 5 Alicante Institute for Health and Biomedical Research (ISABIAL), 03010 Alicante, Spain

**Keywords:** Adult stem cells, Mesenchymal stem cells, Neuronal differentiation, Transdifferentiation, Nucleus, Nuclear positioning

## Abstract

**Background:**

The neuronal transdifferentiation of adult bone marrow cells (BMCs) is still considered an artifact based on an alternative explanation of experimental results supporting this phenomenon obtained over decades. However, recent studies have shown that following neural induction, BMCs enter an intermediate cellular state before adopting neural-like morphologies by active neurite extension and that binucleated BMCs can be formed independent of any cell fusion events. These findings provide evidence to reject the idea that BMC neural transdifferentiation is merely an experimental artifact. Therefore, understanding the intermediate states that cells pass through during transdifferentiation is crucial given their potential application in regenerative medicine and disease modelling.

**Methods:**

In this study, we examined the functional significance of the variety of morphologies and positioning that cell nuclei of human bone marrow-derived mesenchymal stem cells (hBM-MSCs) can adopt during neural-like differentiation using live-cell nuclear fluorescence labelling, time-lapse microscopy, and confocal microscopy analysis.

**Results:**

Here, we showed that after neural induction, hBM-MSCs enter an intermediate cellular state in which the nuclei are able to move within the cells, switching shapes and positioning and even generating cellular protrusions as they attempt to contact the cells around them. These findings suggest that changes in nuclear positioning occur because human cell nuclei somehow sense their environment. In addition, we showed the process of direct interactions between cell nuclei, which opens the possibility of a new level of intercellular interaction.

**Conclusions:**

The present study advances the understanding of the intermediate stage through which hBM-MSCs pass during neural transdifferentiation, which may be crucial to understanding the mechanisms of these cell conversion processes and eventually harness them for use in regenerative medicine. Importantly, our study provides for the first time evidence that the nuclei of hBM-MSC-derived intermediate cells somehow sense their environment, generating cellular protrusions to contact other cells. In summary, human mesenchymal stromal cells could not only help to increase our understanding of the mechanisms underlying cellular plasticity but also facilitate the exact significance of nuclear positioning in cellular function and in tissue physiology.

**Supplementary Information:**

The online version contains supplementary material available at 10.1186/s13287-024-03638-y.

## Introduction

Since the first observation of a nucleus in 1700 [1], our knowledge of nuclear composition, organization, and positioning has continuously evolved [2–4]. Most textbooks depict the nucleus as a spherical or ovoid object at the center of the cell. However, different cell types have very different nuclear shapes, and the position of nuclei varies dramatically from this simple view [2, 3, 5]. Although the cell nucleus has always been considered the largest and most rigid organelle of eukaryotic cells, emerging views of the nucleus indicate a more dynamic organelle than expected [6].

There is increasing evidence that nuclei are frequently asymmetrically positioned depending on cell type, developmental stage, migratory state, and differentiation status [2, 7–10]. It has been reported that the position of the nucleus contributes to cell mechanics, such as gene regulation through relative genome segregation and the organization of cells within tissues [2, 9–11]. Furthermore, it is important to note that changes in nuclear morphology and positioning are often associated with cellular dysfunction and disease [2, 12, 13]. Therefore, the nucleus must be considered not only as the primary site for the storage of genetic material and gene transcription but also as a fundamental mechanical component of the cellular structure [6]. Despite these advances, the exact significance of nuclear positioning in cellular function and tissue physiology is still far from being clearly understood [2, 5, 7].

In our laboratory, we focus on the differentiation of human mesenchymal stem cells (hMSCs) to generate a neuronal lineage. hMSCs are considered promising candidates for cell-based regenerative medicine due to their self-renewal capacity, multilineage differentiation potential, trophic effects and immunomodulatory properties [14, 15]. Controlled neural differentiation of hMSCs could therefore become an important source of cells for cell therapy of neurodegenerative diseases, as autologous adult hMSCs are easily harvested and effectively expanded [16–18].

Over the past two decades, it has been reported that bone marrow-derived cells (BMDCs) and hMSCs can be induced to overcome their mesenchymal fate and differentiate into neural cells, both in vitro [17–25] and in vivo [26–32], a phenomenon known as transdifferentiation. The term transdifferentiation refers to the conversion of one mature cell type into another cell of different blastodermic origin [33, 34]. Such interconversions may involve regression into an intermediate step before cells differentiate into a new blastodermic potential and mature phenotype, or they may occur directly in a process that bypasses such intermediate phenotypes [33, 34]. The actual occurrence of neuronal transdifferentiation of BMDCs and MSCs is currently much debated because the findings and their interpretation have been questioned. The main argument against these observations in culture studies is that MSCs rapidly adopt neural-like morphologies by retraction of the cytoplasm rather than by active neurite extension [35–38]. The in vivo neural transdifferentiation of BMDCs and hMSCs has also been questioned, as cell fusion could explain the development of new cell types that are misinterpreted as transdifferentiated cells [39].

In a previous publication [40], we showed that when human bone marrow-derived MSCs (hBM-MSCs) were exposed to neural induction medium, they rapidly reshaped from a flat to a spherical morphology. Subsequently, hBM-MSCs could maintain the spherical morphology or adopt a new one; they gradually adopted a neural-like morphology through active neurite extension or re-differentiated back to the mesenchymal fate. Furthermore, we found that hBM-MSCs can rapidly and repeatedly switch lineages without cell division. Our results provide evidence that the differentiation of hBM-MSCs into neural-like cells requires a transition through a transient and characterized intermediate state of hBM-MSCs (hBM-MSC-derived intermediate cells) and provide a stronger basis for rejecting the idea that the rapid acquisition of a neural-like morphology during MSC transdifferentiation is merely an artifact.

This previous work also highlights that nuclear remodelling occurs during the in vitro neural-like differentiation of hBM-MSCs. We found that nuclei in hBM-MSC-derived intermediate cells moved within the cell, adopting different morphologies, and even forming two nuclei connected by an internuclear bridge. These nuclear movements generated cellular protrusions that appeared and disappeared from the surface of hBM-MSC-derived intermediate cells. The hBM-MSC-derived intermediate cells positioned their nucleus at the front of the cell during migration. Our results showed that binucleated hBM-MSCs can be formed during neural-like differentiation independent of any cell fusion, providing evidence that transdifferentiation may also be the mechanism behind the presence of gene-marked binucleated neurons after gene-marked bone marrow-derived cell transplantation. Notably, binucleated and polymorphic nuclear cells have been detected in various parts of the nervous system, including adult neurogenic niches [41–45].

Taken together, these findings suggest that to date, there is no conclusive evidence to continue to consider neuronal transdifferentiation of BMDCs and MSCs as a simple experimental artifact, since it recapitulates some structural steps described in vivo [33, 34]. Therefore, future studies are needed to understand the mechanisms of these cell conversion processes and eventually harness them for use in regenerative medicine. In the present study, we investigated the sequence of biological events during neural-like differentiation of hBM-MSCs using live-cell nuclear fluorescence labelling and time-lapse microscopy to understand why the nuclei of hBM-MSC-derived intermediate cells move within the cell, generating the cellular protrusions that appear and disappear from the surface.

## Methods

### Isolation and culture of hBM-MSCs

A standard protocol for the isolation and expansion of hMB-MSCs was used as previously described [40]. Bone marrow aspirates were obtained by percutaneous direct aspiration from the iliac crest of 5 healthy volunteers at University Hospital Virgen de la Arrixaca (Murcia, Spain). Bone marrow was collected with 20 U/ml sodium heparin, followed by Ficoll density gradient-based separation by centrifugation at 540 g for 20 min. After, the mononuclear cell fraction was collected, washed twice with Ca^2+^/Mg^2+^-free phosphate buffered saline (PBS) (Gibco Invitrogen) and seeded into 175-cm2 culture flasks (Nunc, Thermo Fisher Scientific) at a cell density of 1.5 × 10^5^ cells/cm^2^ in serum-containing media (designated as the basal media), composed of DMEM low glucose medium (Thermo Fisher Scientific) supplemented with 10% fetal bovine serum (FBS; Lonza), 1% GlutaMAX (Thermo Fisher Scientific), nonessential amino acid solution (Sigma‒Aldrich) and 1% penicillin/streptomycin (Thermo Fisher Scientific). After 3 days of culture at 37 °C and 7% CO_2_, nonadherent cells were removed, and fresh complete medium was added. Culture media were renewed every 2 days, and the isolated hMB-MSCs were passaged when cultures were 70–80% confluent. All studies were performed using hMB-MSCs expanded within culture passages 3–4.

### Expression vectors and cell transfection

The expression vectors used in the present study were H2B-eGFP, a gift from Geoff Wahl (Addgene plasmid # 11680; http://n2t.net/addgene:11680; RRID:Addgene_11680) [46]. A standard protocol for transfecting MSCs was used as previously described [40]. Isolated hMB-MSCs were transfected using the Gene Pulser-II Electroporation System (Bio-Rad Laboratories). Electroporation was performed in a sterile cuvette with a 0.4-cm electrode gap (Bio-Rad Laboratories) using a single pulse of 270 V and 500 μF. Plasmid DNA (5 μg) was added to 1.5 × 10^6^ viable hMB-MSCs in 0.2 ml of DMEM low glucose medium (Thermo Fisher Scientific) before electrical pulsing.

### Time-lapse microscopy of histone H2B-GFP-expressing hBM-MSCs cultured in neural induction media

We used μ-Dish 35 mm, high Grid-500 (Ibidi) for live cell imaging. Histone H2B-GFP-transfected hBM-MSCs were plated onto collagen IV (Sigma‒Aldrich)-coated plastic or glass coverslips. To induce neural differentiation, cells at passages 3–4 were allowed to adhere to the plates overnight. Basal media was removed the following day, and the cells were cultured for 2 days in serum-free media (designated as the neural basal media) consisting of Dulbecco’s modified Eagle’s medium/F12 (DMEM/F12 Glutamax, Gibco) supplemented with N2-supplement (R&D systems), 0.6% glucose (Sigma‒Aldrich), 5 mM HEPES (Sigma‒Aldrich), 0.5% human serum albumin (Sigma‒Aldrich), 0.0002% heparin (Sigma‒Aldrich), nonessential amino acid solution (Sigma‒Aldrich) and 100 U/ml penicillin‒streptomycin (Sigma‒Aldrich). On day 3, the cells were cultured in neural induction media consisting of neural basal media supplemented with 500 nM retinoic acid (Sigma‒Aldrich) and 1 mM dibutyryl cAMP (Sigma‒Aldrich). Time-lapse analysis was carried out using a Widefield Leica Thunder-TIRF imager microscope. We performed time-lapse microscopy within the first 70 h after neural induction media was added directly to the cells. Time-lapse images were obtained with a 40X objective every 10 min. During imaging, cells were enclosed in a chamber maintained at 37 °C under a humidified atmosphere of 5% CO2 in air. Data are representative of ten independent experiments.

### Immunocytochemistry

A standard immunocytochemical protocol was used as previously described [40, 45, 47–49]. Histone H2B-GFP-transfected hBM-MSCs were plated onto collagen IV (Sigma‒Aldrich)-coated plastic or glass coverslips and maintained in neural induction media. Cells were rinsed with PBS and fixed in freshly prepared 4% paraformaldehyde (PFA; Sigma‒Aldrich). Fixed cells were blocked for 2 h in PBS containing 10% normal horse serum (Gibco) and 0.25% Triton X-100 (Sigma) and incubated overnight at 4 °C with antibodies against β-III-tubulin (TUJ1; 1:500, Covance), fibrillarin (1/300, Abcam) and lamin A/C (1/300, GeneTex) in PBS containing 1% normal horse serum and 0.25% Triton X-100. The next day, the cells were rinsed and incubated with secondary antibodies conjugated with Alexa Fluor® 488 (anti-rabbit; 1:500, Molecular Probes) and Alexa Fluor® 594 (anti-mouse; 1:500, Molecular Probes). Cell nuclei were counterstained with DAPI (0.2 mg/ml in PBS, Molecular Probes). Alexa Fluor 488® phalloidin (Molecular Probes) was used to selectively stain F-actin. Data are representative of ten independent experiments per condition.

### Images and data analyses

Photographs of visible and fluorescent stained samples were obtained using a Widefield Leica Thunder-TIRF imager microscope equipped with a digital camera or a Leica TCS-SP8 confocal laser scanning microscope. We used Leica Application Suite X and Imaris software for image analysis and Filmora Video Editor software for video editing. Photoshop software was used to improve the visibility of fluorescence images without altering the underlying data. Data are representative of ten independent experiments per condition and are expressed as the mean ± SD.

## Results

### Characterization of hBM-MSC cultures

In a previous publication, we showed that hBM-MSCs did not express hematopoietic lineage markers such as CD45, CD14, CD34 and CD20 and were positive for CD90, CD105, and CD73, thereby demonstrating a characteristic immunophenotype of hMSCs [40]. Under proliferation conditions, hBM-MSCs displayed a flat, fibroblast-like morphology with β-III-tubulin microtubules and actin microfilaments oriented parallel to the longitudinal axis of the cell (Additional file 1: Fig. S1A). During interphase, hBM‑MSCs displayed a flattened, ellipsoidal nucleus, often located in the center of the cell and with a nuclear volume of approximately 419′30 ± 106′38 μm^3^ (Additional file 1: Fig. S1B). The dynamic localization of the nuclear lamina was analysed by immunostaining for lamin A/C, a nuclear lamina component [50], and the dynamic localization of the nucleoli was analysed by immunostaining for fibrillarin, the main component of the active transcription centers [51]. A speckled pattern was observed distributed throughout the nucleus with heterogeneity in the number, size, and distribution of the fibrillarin-positive specks (Additional file 1: Fig. S1C). Laser scanning confocal microscopy revealed that the inner surface of the nuclear envelope is lined by the nuclear lamina (Additional file 1: Fig. S1D).

### Characterization of hBM-MSC-derived intermediate cells

In a previous publication [40], we showed that following neural induction, hBM-MSCs rapidly reshaped from a flat to a spherical morphology (hBM-MSC intermediate cells). Subsequently, we observed that hBM-MSC-derived intermediate cells can preserve their spherical shape, change to that of neural-like cells through active neurite extension or revert back to their mesenchymal morphology. In this study, we focused on hBM-MSC-derived intermediate cells that can maintain their spherical shape for several days without assuming new fates. To better understand why the nuclei of hBM-MSC-derived intermediate cells move within the cell to generate cellular protrusions, we performed time-lapse microscopy and immunocytochemical analyses of histone H2B-GFP-transfected hBM-MSCs within the first 70 h of neural induction. The histone–GFP fusion protein enables sensitive analysis of chromosome dynamics in living mammalian cells without perturbing intracellular structures or cell cycle control [46]. As a negative control, H2B-GFP-transfected hBM-MSCs were cultured in a non-neuronal induction medium (basal media). The time-lapse experiments were performed using a higher magnification objective and a shorter image capture interval than previously published experiments [40].

**Fig. 1:**
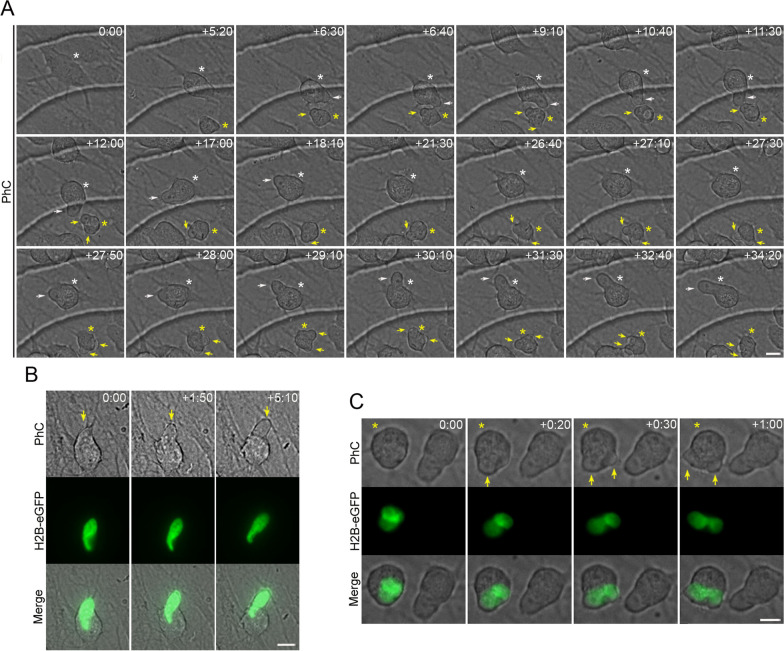
Nuclear movement generated cellular protrusions that appeared and disappeared from the surface of hBM-MSC-derived intermediate cells. **A** Time-lapse imaging showed that when hBM-MSCs were exposed to neural induction medium, they rapidly reshaped from a flat to a spherical morphology. Subsequently, we observed hBM-MSC-derived intermediate cells (white asterisk) in which nuclear movements generate only one cell protrusion (white arrow) and hBM-MSC-derived intermediate cells (yellow asterisk) in which nuclear movements alternately generate one or two cellular protrusions (yellow arrows). **B** We found that when an hBM-MSC-derived intermediate cell has a nucleus without lobes, its movement within the cell generates only one cell protrusion (yellow arrow). **C** However, if the hBM-MSC-derived intermediate cell has a lobed nucleus, it will generate one or two cellular protrusions depending on how it moves within the cell (yellow arrows). Scale bar: 10 μm. PhC: Phase-contrast photomicrographs. The number at the top indicates the time since the time-lapse image began. Elapsed time is displayed in the format (hours:minutes)

Time-lapse imaging revealed that H2B-GFP-transfected hBM-MSCs do not spontaneously differentiate into neural-like cells when cultured in a non-neuronal induction medium. Furthermore, we observed that the nuclei of transfected and non-transfected cells did not change shape or generate cellular protrusions (Additional file 2: Fig. S2, Additional file 3: Movie S1). However, we noted that when hBM-MSCs were exposed to neural induction medium, they rapidly reshaped from a flat to a spherical morphology (Fig. 1A; Additional file 4: Movie S2). We then observed hBM-MSC-derived intermediate cells in which nuclear movements generated only one cell protrusion (Fig. 1A, white arrow; Additional file 4: Movie S2) and hBM-MSC-derived intermediate cells in which nuclear movements alternately generated one or two cellular protrusions (Fig. 1A, yellow arrows; Additional file 4: Movie S2). We found that when hBM-MSC-derived intermediate cells have a nucleus without lobes, their movement within the cell generates only one cell protrusion (Fig. 1B; Additional file 5: Movie S3). However, if the hBM-MSC-derived intermediate cell has a lobed nucleus, it will generate one or two cellular protrusions depending on how it moves within the cell (Fig. 1C).

**Fig. 2:**
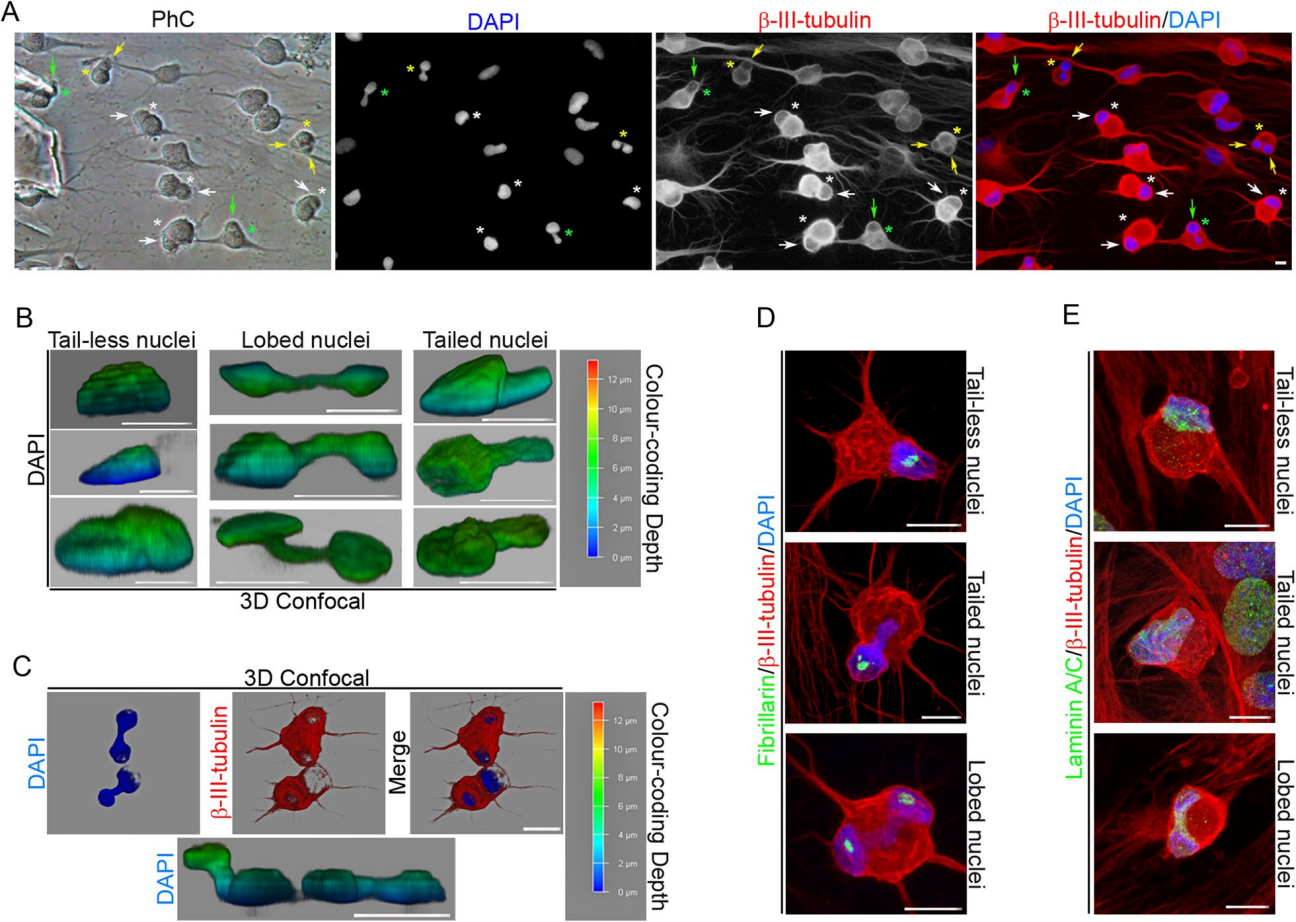
Characterization of hBM-MSC-derived intermediate cell nuclei. **A** Immunocytochemical analysis revealed that hBM-MSC-derived intermediate cells primarily have three different nuclear morphologies: Tail-less nuclei (white asterisk), tailed nuclei (green asterisk) and lobed nuclei (yellow asterisk). Tail-less and tailed nuclei movements generate only one cell protrusion (white and green arrows, respectively). However, lobed nuclei movements generate one or two cellular protrusions depending on how they move within the cell (yellow arrows). Confocal microscopy analysis and 3D reconstruction revealed that there are small variations in both shape and size within the three types of nuclear morphology (**B**) and that the lobes of the lobed nuclei can be located in different *z*-planes. **C** Immunocytochemical analysis revealed that tail-less nuclei and tailed nuclei contained one or two fibrillarin-positive specks, while lobed nuclei contained one or two fibrillarin-positive specks in each lobe. **D** The inner surface of the nuclear envelope is lined by the nuclear lamina (**E**). Scale bar: 10 μm. PhC: Phase-contrast photomicrographs

Although the cell nuclei switch their morphologies while moving, time-lapse imaging and immunocytochemical analysis revealed that hBM-MSC-derived intermediate cells have three main different nuclear morphologies: tail-less nuclei (Fig. 2A, white asterisk), tailed nuclei (Fig. 2A, green asterisk) and lobed nuclei (Fig. 2A, yellow asterisk). Tail-less and tailed nuclei movements generate only a single cell protrusion (Fig. 2A, white and green arrows, respectively). However, as mentioned above, lobed nucleus movements generate one or two cellular protrusions depending on how they move within the cell (Fig. 2A, yellow arrows). Confocal microscopy analysis and 3D reconstruction revealed that there were small variations in both shape and size within the three types of nuclear morphology (Fig. 2B). Tail-less nuclei, tailed nuclei and lobed nuclei have a volume of approximately 327′11 ± 94′19 μm^3^, 306′89 ± 16′50 μm^3^ and 361′75 ± 147′44 μm^3^, respectively. It is important to note that confocal microscopic analysis and 3D reconstruction also revealed that the lobes of the lobed nuclei can be located in different z-planes (Fig. 2C). We observed that tail-less nuclei and tailed nuclei contained one or two fibrillarin-positive specks, whereas lobed nuclei contained one or two fibrillarin-positive specks in each lobe (Fig. 2D). No positive fibrillarin specks were detected either in the tail of the tailed nuclei or in the region of nucleoplasm connecting each lobe of lobed nuclei. Laser scanning confocal microscopy also revealed that the inner surface of the nuclear envelope is lined by the nuclear lamina (Fig. 2E).

Time-lapse imaging also revealed that changes in nuclear positioning are not attributable to the cell body undergoing rotation or attempted migration, as it is possible to observe non-migratory cells in which tail-less nuclei (Additional file 6: Fig. S3A; Additional file 7: Movie S4), tailed nuclei (Additional file 6: Fig. S3B; Additional file 8: Movie S5) and lobed nuclei (Additional file 6: Fig. S3C; Additional file 9: Movie 6) can change positions, while cell body projections remain in the same cell positions.

**Fig. 3:**
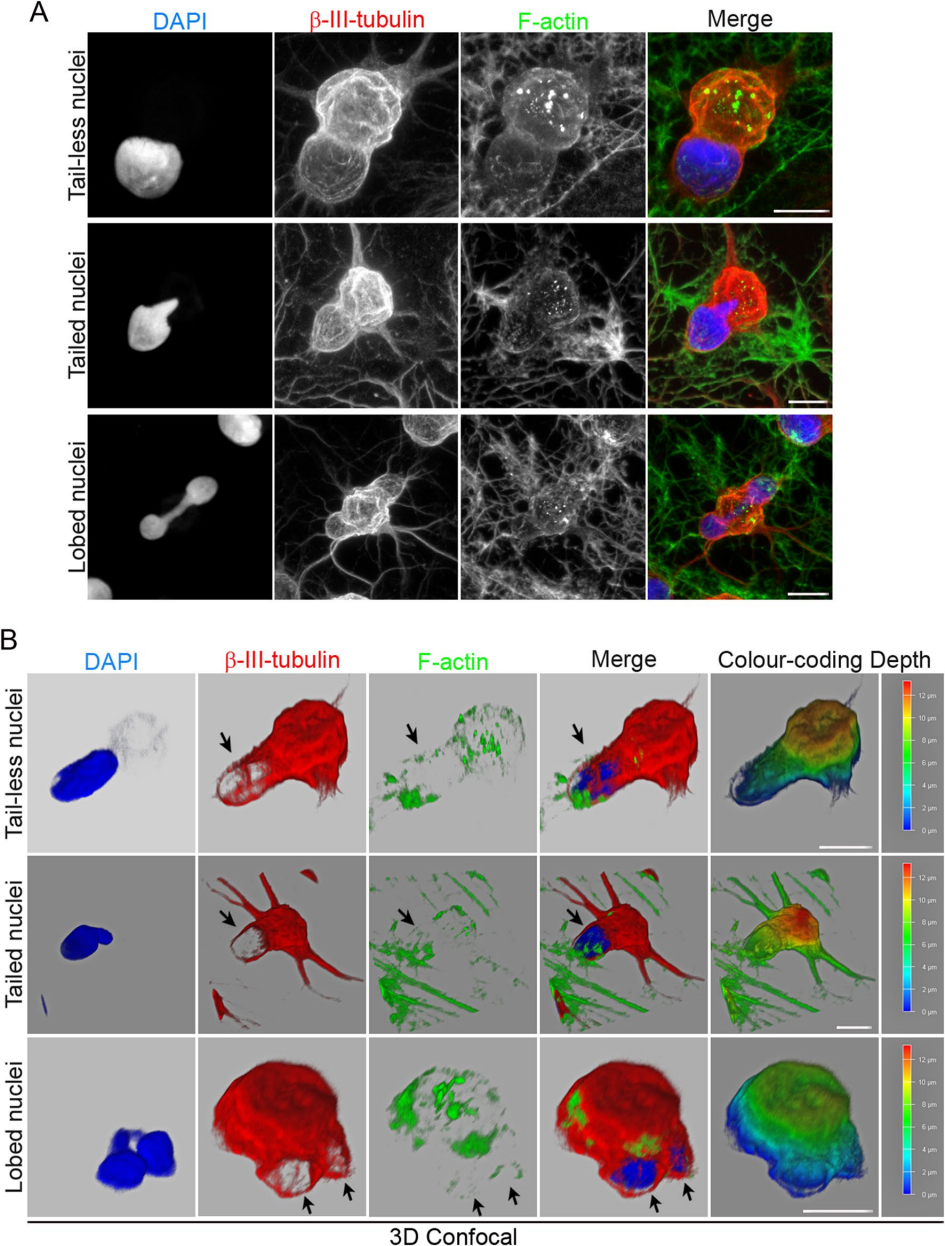
Cytoskeletal organization of hBM-MSC-derived intermediate cells. **A** Immunocytochemical analysis revealed that actin microfilaments and β-III tubulin microtubules are no longer oriented parallel to the longitudinal axis of the hBM-MSC-derived intermediate cells. **B** Confocal microscopy analysis and 3D reconstruction revealed that cell protrusions are almost devoid of actin microfilaments and that the β-III tubulin protein is concentrated at the cell protrusion rim (black arrows). Scale bar: 10 μm

Immunocytochemical analysis revealed that actin microfilaments and β-III tubulin microtubules were no longer oriented parallel to the longitudinal axis of the hBM-MSC-derived intermediate cells (Fig. 3A). Furthermore, confocal microscopy analysis and 3D reconstruction revealed that cell protrusions are almost devoid of actin microfilaments and that the β-III tubulin protein is concentrated at the cell protrusion rim (Fig. 3B, black arrows). It has been reported that direct connections between the actin cytoskeleton and the nucleus govern nuclear positioning and nuclear movement during cell polarization and migration [2, 7, 8]. Therefore, it would be interesting to examine the role of the cytoskeleton and/or nucleoskeleton in the formation and movement of the nuclei of hBM-MSC-derived intermediate cells to understand whether changes in morphology and nuclear positioning are attributable to chromatin movement or to the role of cytoskeleton and/or nucleoskeleton.

### Nuclear remodelling

To further understand how the three different types of nuclei that move within the hBM-MSC-derived intermediate cells are formed, we examined the sequence of biological events during neural-like differentiation of histone H2B-GFP-transfected hBM-MSCs using time-lapse microscopy. It is important to note that the hBM-MSCs did not differentiate at the same time or rate, so the cell culture simultaneously contained hBM-MSCs at different stages of neural-like differentiation.

**Fig. 4:**
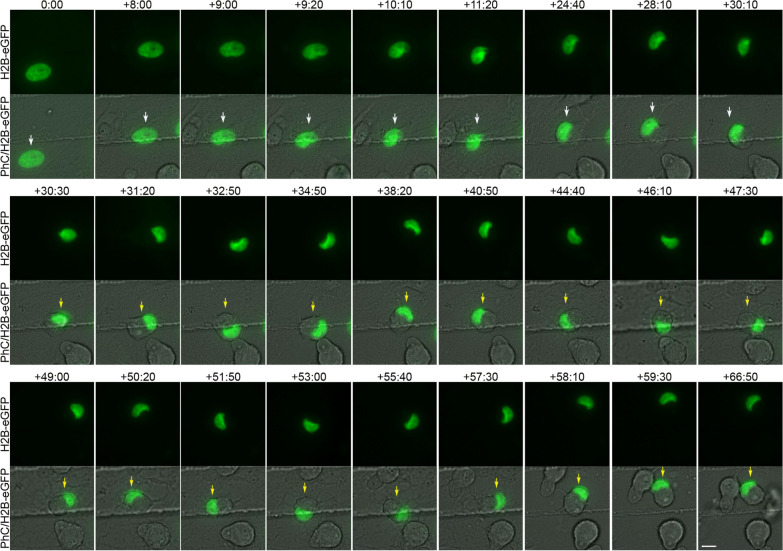
Tail-less nuclei formation. Time-lapse imaging revealed that tail-less nuclei are formed by a single nuclear remodelling that occurs as the cell reshapes from a flat to a spherical morphology, positioning the nucleus in a peripheral position within the cell (white arrows). Subsequently, the tail-less nucleus began to move within the hBM-MSC-derived intermediate cells (yellow arrows). Scale bar: 10 μm. The number at the top indicates the time since the time-lapse image began. Elapsed time is displayed in the format (hours:minutes)

Time-lapse imaging revealed that there are also variations in the form and time at which cell nuclei adopt these different nuclear morphologies (Additional file 10: Movie S7, Additional file 11: Movie S8, Additional file 12: Movie S9, Additional file 13: Movie S10, Additional file 14: Movie S11, Additional file 15: Movie S12, Additional file 16: Movie S13, Additional file 17: Movie S14, Additional file 18: Movie S15). Although future studies are needed to determine all the possible different nuclear remodelling sequences that occur when hBM-MSCs reshape from a flat to a spherical morphology, below, we have described some examples of the formation of each of the three types of nuclei that move within hBM-MSC-derived intermediate cells. We observed that tail-less nuclei are formed by a single nuclear remodelling that occurs as the cell reshapes from a flat to a spherical morphology, positioning the nucleus in a peripheral position within the cell (Fig. 4, white arrows; Additional file 10: Movie S7, Additional file 11: Movie S8). The duration of this particular process is approximately 30 h. Subsequently, the tail-less nucleus began to move within the hBM-MSC-derived intermediate cells. (Fig. 4, yellow arrows; Additional file 10: Movie S7, Additional file 11: Movie S8).

**Fig. 5:**
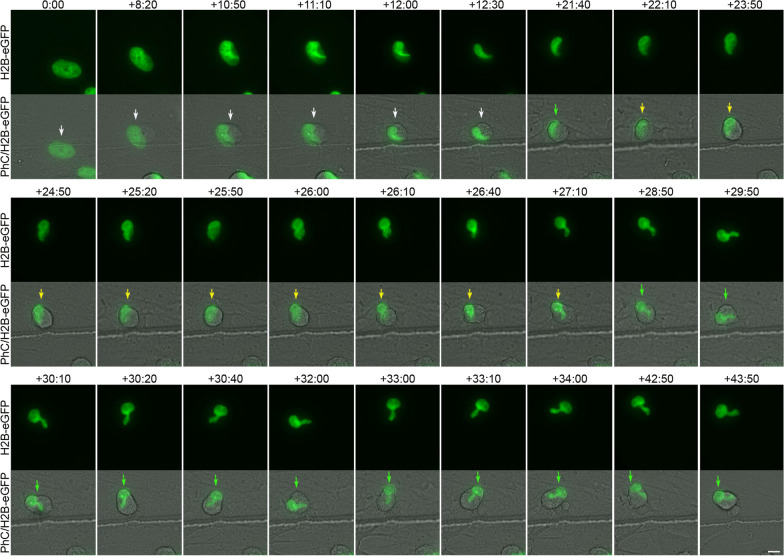
Tailed nuclei formation by two nuclear remodelling sequences. Time-lapse images show that first, a nuclear remodelling sequence occurs as the cell reshapes from a flat to a spherical morphology, positioning the nucleus in a peripheral position within the cell (white arrows). Next, the cell nucleus moves (green arrows) and undergoes a second nuclear sequence in which a tailed process in the nucleus is formed (yellow arrows). Finally, the tailed nucleus began to move within the hBM-MSC-derived intermediate cells (green arrows). Scale bar: 10 μm. The number at the top indicates the time since the time-lapse image began. Elapsed time is displayed in the format (hours:minutes)

Time-lapse images also showed that tailed nuclei are formed by one (Additional file 12: Movie S9) or two nuclear remodelling sequences. Below, we show an example of the formation of a tailed nucleus generated by two nuclear remodelling sequences (Fig. 5; Additional file 13: Movie S10, Additional file 14: Movie S11). We found that first, a nuclear remodelling sequence occurs as the cell reshapes from a flat to a spherical morphology, positioning the nucleus in a peripheral position within the cell (Fig. 5, white arrows; Additional file 13: Movie S10, Additional file 14: S11). The duration of this particular process is approximately 12 h. Subsequently, the cell nucleus moves (Fig. 5, green arrows; Additional file 13: Movie S10, Additional file 14: Movie S11) and undergoes a second nuclear remodelling sequence in which a tailed process in the nucleus is formed (Fig. 5, yellow arrows; Additional file 13: Movie S10, Additional file 14: Movie S11). The duration of this particular process is approximately 5 h. Finally, the tailed nucleus began to move within the hBM-MSC-derived intermediate cells. (Fig. 5, green arrows; Additional file 13: Movie S10, Additional file 14: Movie S11). Time-lapse imaging also revealed that as the tailed nuclei move within the cell, the tails can switch shape and size (Additional file 19: Fig. S4A; Additional file 15: Movie 12) and even appear to move in different z-planes (Additional file 19: Fig. S4B; Additional file 13: Movie S10, Additional file 14: Movie S11). Future analyses will be needed to determine whether the nuclei use the tails to stabilize their position within the cell.

**Fig. 6:**
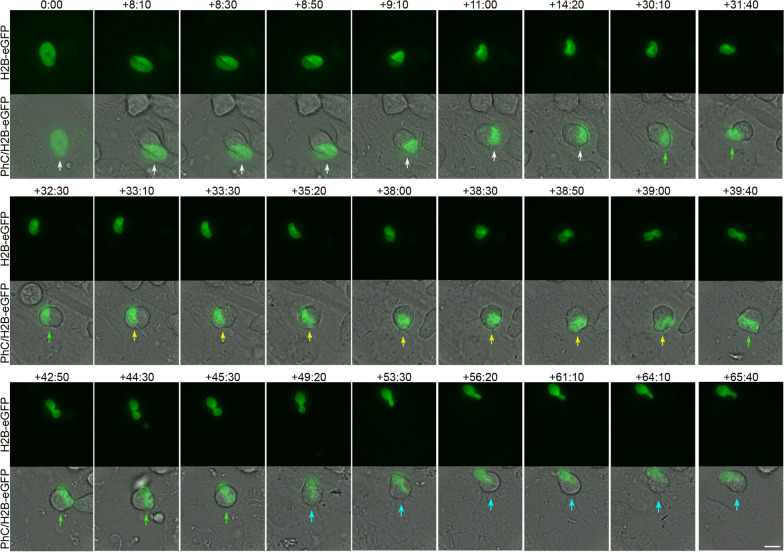
Lobed nuclei formation by two nuclear remodelling sequences. Time-lapse imaging showed that first, a nuclear remodelling sequence occurs as the cell reshapes from a flat to a spherical morphology, positioning the nucleus in a peripheral position within the cell (white arrows). Subsequently, the cell nucleus moves (green arrows) and undergoes a second nuclear sequence in which a lobed nucleus is formed (yellow arrows). Afterwards, the lobed nuclei began to move within the hBM-MSC-derived intermediate cells (green arrows). Finally, we also noted that lobed nuclei can switch shape while moving within the cell, becoming tailed nuclei (blue arrows). Scale bar: 10 μm. The number at the top indicates the time since the time-lapse image began. Elapsed time is displayed in the format (hours:minutes)

Time-lapse imaging also revealed that lobed nuclei are formed by one (Additional file 15: Movie S12) or two nuclear remodelling sequences. Below, we show you an example of the formation of a lobed nucleus generated by two nuclear remodelling sequences (Fig. 6; Additional file 16: Movie S13, Additional file 17: Movie S14). We found that first, a nuclear remodelling sequence occurs as the cell reshapes from a flat to a spherical morphology, positioning the nucleus in a peripheral position within the cell (Fig. 6, white arrows; Additional file 16: Movie S13, Additional file 17: Movie S14). The duration of this particular process is approximately 14 h. Subsequently, the cell nucleus moves (Fig. 6, green arrows; Additional file 16: Movie S13, Additional file 17: Movie S14) and undergoes a second nuclear remodelling sequence in which a lobed nucleus is formed (Fig. 6, yellow arrows; Additional file 16: Movie S13, Additional file 17: Movie S14). The duration of this particular process is approximately 5 h. The lobed nuclei then began to move within the hBM-MSC-derived intermediate cells (Fig. 6, green arrows; Additional file 16: Movie S13, Additional file 17: Movie S14). Finally, we also found that lobed nuclei can switch shape while moving within the cell, becoming tailed nuclei (Fig. 6, blue arrows; Additional file 16: Movie S13, Additional file 17: Movie S14).

As mentioned above, confocal microscopy analysis and 3D reconstruction revealed that the lobes of the lobed nuclei can be located in different z-planes (Fig. 2C). This result, together with the time-lapse noted in Fig. 1A and Additional file 4: Movie S2, suggests that each lobule of the lobed nuclei can move in different z-planes. Quantitative analysis of time-lapse imaging revealed that the nuclear speed oscillated between 0.1 and 1.3 μm/min, with an average speed of 0.66 ± 0.45 μm/min. These findings are consistent with previous studies that reported the physical characteristics of typical nuclear movement in different cell types [2]. Nuclear movement can generate cellular protrusions that extend up to a length similar to the cell diameter (Fig. 1,3; Additional file 4: Movie S2, Additional file 5: Movie S3).

### hBM-MSC-derived intermediate cells use their cell nuclei to interact with other cells

**Fig. 7:**
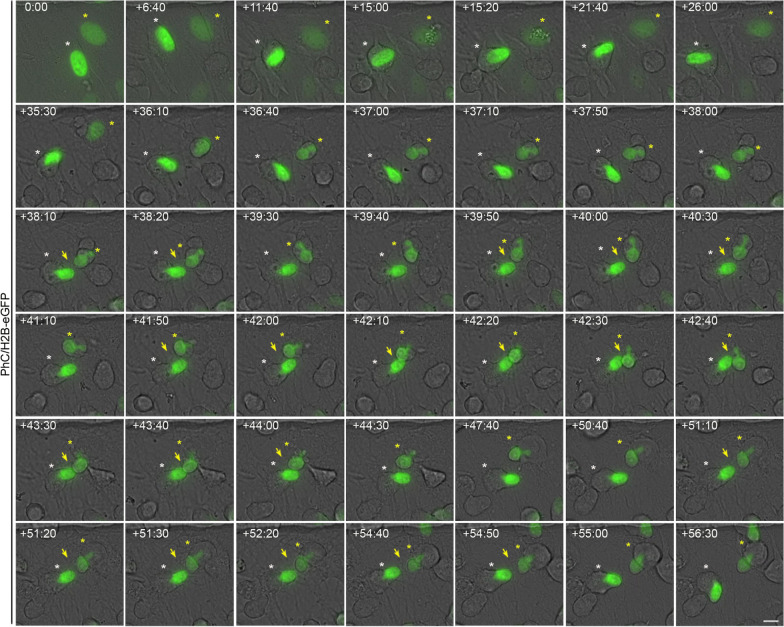
hBM-MSC-derived intermediate cells interact with each other through their cell nuclei. Time-lapse imaging revealed that hBM-MSC-derived intermediate cell nuclei move within the cell, generating cellular protrusions as they attempt to contact the cells around them, mainly observing interactions between the nuclei (arrows). Scale bar: 10 μm. The number at the top indicates the time since the time-lapse image began. Elapsed time is displayed in the format (hours:minutes)

**Fig. 8:**
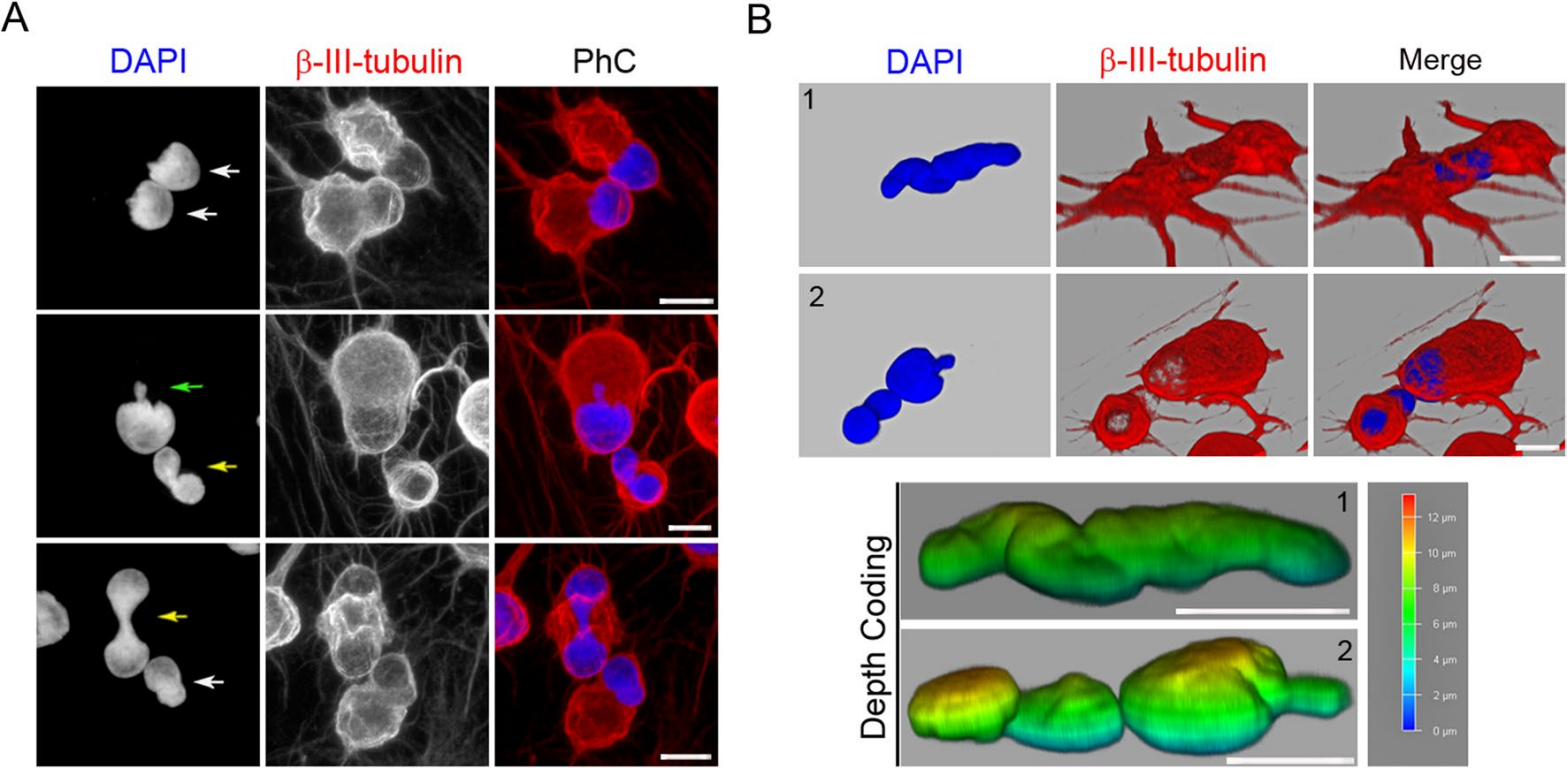
Tail-less nuclei, tailed nuclei and lobed nuclei interact with each other. Confocal microscopy analysis (**A**) and 3D reconstruction (**B**) revealed that tail-less nuclei (white arrow), tailed nuclei (green arrow) and lobed nuclei (yellow arrow) interact each other. Scale bar: 10 μm

Time-lapse imaging revealed that hBM-MSC-derived intermediate cell nuclei move within the cell, generating cellular protrusions as they attempt to contact surrounding cells (Figs. 1, 4, 6; Additional file 4: Movie S2, Additional file 5: Movie S3, Additional file 8: Movie S5, Additional file 10: Movie S7, Additional file 16: Movie S13). Furthermore, we observed that interactions occurred between the cell nuclei of hBM-MSC-derived intermediate cells, even for several hours (Fig. 7; Additional file 18: Movie S15). Confocal microscopy analysis (Fig. 8A) and 3D reconstruction (Fig. 8B) revealed that tail-less nuclei, tailed nuclei and lobed nuclei interact with each other. We note that lobed nuclei can interact with other cells through one lobe or both simultaneously or successively (Fig. 1A; Additional file 3: Movie 1). Taken together, these findings suggest that changes in nuclear positioning occur because cell nuclei somehow sense their surroundings.

## Discussion

The fate of adult cells was thought to be restricted to their tissue blastodermic of origin [52]. However, there is now a large body of evidence suggesting that under physiological conditions and certain experimental conditions, adult cells may be more plastic than we previously thought in that they can become cells of unrelated lineages, a phenomenon known as transdifferentiation sensu stricto [33, 34, 53, 54]. The most evident transdifferentiation events are epithelial-mesenchymal transition (EMT) and its reverse process mesenchymal-epithelial transition (MET) [55–57]. These processes are associated with implantation, embryo formation, organ development and wound healing [55–57]. It is important to note that transdifferentiation events have been implicated in pathological conditions, such as organ fibrosis, and in cancer, where they contribute to tumor progression and metastasis [55–57]. In the process of transdifferentiation, cells pass through intermediate states that are not well understood [33, 55–58]. Given the potential application of this cell conversion process, not only in developmental and cancer studies but also in regenerative medicine, a better understanding of intermediate states is crucial to avoid uncontrolled conversion or proliferation, which poses a risk to patients [33, 55–58].

Over the last two decades, it has been reported that BMDCs and hMSCs can be induced to overcome their mesenchymal fate and transdifferentiate into neural cells, both in vitro [17–25] and in vivo [26–32]. However, the neuronal transdifferentiation of BMDCs and MSCs is still considered to be merely an artifact. The main argument against these observations in culture studies is that MSCs rapidly adopt neuronal-like morphologies by retraction of the cytoplasm rather than by active neurite extension [35–38]. The main argument against neuronal transdifferentiation of BMDCs and hMSCs in vivo is that cell fusion could explain the development of new cell types that are misinterpreted as transdifferentiated cells [39].

In previous publications, we have shown that MSCs isolated from adult human tissues can differentiate into neural-like cells, both in vitro and in vivo [40, 45, 47–49]. In vitro, hMSCs differentiate into neural-like cells based on cellular morphology and neural marker expression [40, 45, 47, 49]. In vivo, hMSCs-derived neural-like cells survived, migrated and expressed neural markers after being grafted to the adult mouse brain. Importantly, the hMSCs-derived neural-like cells located in the neural stem cell niches, such as the ventricular-subventricular zone of the anterolateral ventricle wall and the subgranular zone of the hippocampal dentate gyrus, show neural stem morphology [47].

In vitro studies also showed that when hBM-MSCs were exposed to neural induction medium, they rapidly reshaped from a flat to a spherical morphology [40]. Subsequently, hBM-MSCs could maintain the spherical morphology or adopt a new morphology; they gradually adopted a neural-like morphology through active neurite extension or re-differentiated back to the mesenchymal fate. Furthermore, we found that hBM-MSCs can rapidly and repeatedly switch lineages without cell division. These results provide evidence that the differentiation of hBM-MSCs into neural-like cells requires a transition through an intermediate state, as described in natural transdifferentiation processes [33, 34, 55–58]. This previous work also highlights that nuclear remodelling occurs during in vitro neural-like differentiation of hBM-MSCs [40]. We found that nuclei in hBM-MSC-derived intermediate cells moved within the cell, adopting different morphologies and even forming two nuclei connected by an internuclear bridge, independent of any cell fusion.

These results provide a strong basis for rejecting the idea that the rapid acquisition of a neural-like morphology during in vitro MSC transdifferentiation is merely an artifact and provide evidence that transdifferentiation may also be the mechanism behind the presence of gene-marked binucleated neurons after gene-marked bone marrow-derived cell transplantation.

It is important to note that the studies describing the presence of binucleated Purkinje neurons after bone marrow-derived cell transplantation suggest, but do not conclusively demonstrate, that cell fusion is the underlying mechanism to explain the presence of binucleated neurons [39, 59, 60]. Furthermore, binucleated Purkinje neurons are also present in healthy, unmanipulated mice and humans [39, 61]. In addition, many authors have described binucleated neurons in various central and peripheral parts of the nervous system [62–65]. Moreover, many authors have reported that many cultured hippocampal neurons [42] (their Fig. S2) and neural stem cells located in the ventricular-subventricular zone of the anterolateral ventricular wall of the human fetal brain [43] (their Fig. 2C) and adult mouse brain [43, 44] (their Figs. 1E, 4I, 6F, S1B, S2B, S6A and their Fig. 3A, respectively) also have two nuclei connected by an internuclear bridge.

Taken together, these results suggest that to date, there is no conclusive evidence to continue to consider the neuronal transdifferentiation of BMDCs and hMSCs as an artefact. Therefore, future studies are needed to optimize the various neural induction protocols that have been developed for MSCs [66], not only to understand the mechanisms of these cellular conversion processes but also to eventually harness them for use in regenerative medicine [16, 33, 58, 67].

Our current research aims to improve our understanding of what hBM-MSCs look like at this intermediate stage. We wanted to know why the nuclei of hBM-MSC-derived intermediate cells move within the cell and generate the cellular protrusions that appear and disappear from the surface. In this study, we have shown that once hBM-MSCs enter this intermediate stage, the cell nuclei begin to move within the cell and generate cellular protrusions as they try to contact the surrounding cells. Interkinetic nuclear migration (INM) in neural progenitors underlies normal neurogenesis during normal development [68, 69], suggesting that the observed nuclear motility in hBM-MS-derived intermediated cells recapitulates an intermediate stage before deciding the proliferation of differentiation progression in development and transdifferentiation. Importantly, in a previous publication, we observed that the nuclei of hBM-MSCs can also exhibit oscillatory movement along the axis of cell polarity, similar to that described to occur during INM [40] (their Fig. S4 and Supplementary Video S4).

We also found that interactions occur between the cell nuclei of hBM-MSC-derived intermediate cells, even for several hours. These findings suggest that changes in nuclear positioning occur because cell nuclei somehow sense their surroundings. To our knowledge, we have described for the first time the process of direct interactions between cell nuclei, which opens the possibility of a new level of intercellular interaction. During this intermediate phase, nuclear factors may be interchanged by neighboring cells and directly modify transcriptomes. Extensive studies are needed to determine the mechanisms and consequences of this process of direct interactions between cell nuclei in normal and pathologic circumstances.

This work also highlights that cell nuclei moving within hBM-MSC intermediate cells have three main different morphologies: tail-less nuclei, tailed nuclei, and lobed nuclei. We have shown that there are variations in the shape and time at which the nuclei of hBM-MSCs adopt the different nuclear morphologies observed in hBM-MSC-derived intermediate cells. We also found that tail-less and tailed nuclei movements generate only a single cell protrusion when attempting to contact other cells. However, lobed nuclei movements generate one or two cellular protrusions depending on how they move within the cell. Lobed nuclei may interact with other cells through one lobe or both simultaneously or successively. Nevertheless, it is important to note that the three different nuclear morphologies observed in hBM-MSC intermediate cells can be interchanged as the nucleus moves within the cell. Although future experiments will be needed to understand why there are different nuclear morphologies in hBM-MSC-derived intermediate cells and why they are formed in different ways, our results demonstrate that the nucleus should be considered not only as the primary site for the storage of genetic material and the transcription of genes but also as a fundamental mechanical component of the cell.

## Conclusions

In conclusion, our findings enrich the understanding of intermediate states in the neural-like differentiation process of hBM-MSCs and suggest that changes in nuclear positioning occur because human cell nuclei somehow sense their environment. Our results also provide additional evidence that adult cells can assume new fates without asymmetric cell division and lend further support to the notion that MSCs transdifferentiate towards a neural lineage through an intermediate state. Although the neuronal transdifferentiation of BMDCs and MSCs is still considered to be merely an artifact, it is unreasonable to ignore that there is increasing evidence supporting this phenomenon. Human mesenchymal stromal cells could not only help to increase our understanding of the mechanisms underlying cellular plasticity and eventually harness them for use in regenerative medicine but also facilitate an understanding of the mechanisms regulating nuclear structure and dynamics.

### Supplementary Information

**Additional file 1: Figure S1.** Morphology of hBM-MSCs cultured in basal medium. **A** Undifferentiated hBM-MSCs exhibited a fibroblast-like morphology with β-III tubulin microtubules and actin microfilaments oriented parallel to the longitudinal axis of the cell. **B** During interphase, hBM‑MSCs displayed a flattened, ellipsoidal nucleus, often located in the center of the cell. **C** Distribution of fibrillarin-positive specks in the nuclei of undifferentiated hBM-MSCs. **D** Immunocytochemical analysis revealed that the inner surface of the nuclear envelope is lined by the nuclear lamina. Scale bar: 10 μm.

**Additional file 2: Figure S2.** Spontaneous neural-like differentiation was not detected in H2B-GFP-transfected hBM-MSCs without neuronal induction. Time-lapse imaging revealed that H2B-GFP-transfected hBM-MSCs do not spontaneously differentiate into neural-like cells when cultured in a non-neuronal induction medium. The nuclei of the transfected (yellow arrows) and non-transfected cells (white arrows) did not change shape or generate cellular protrusions. Scale bar: 10 μm. The number at the top indicates the time since the time-lapse image began. Elapsed time is displayed in the format (hours:minutes).

**Additional file 3: Movie S1.** Spontaneous neural-like differentiation was not detected in H2B-GFP-transfected hBM-MSCs without neuronal induction. Related to Fig. S2. Time-lapse imaging revealed that H2B-GFP-transfected hBM-MSCs do not spontaneously differentiate into neural-like cells when cultured in a non-neurogenic medium. Furthermore, the nuclei of the transfected (yellow arrows) and non-transfected cells (white arrows) did not change shape or generate cellular protrusions.

**Additional file 4: Movie S2.** Nuclear movement generated cellular protrusions that appeared and disappeared from the surface of hBM-MSC-derived intermediate cells. Related to Fig. 1A. Time-lapse phase-contrast imaging showed that when hBM-MSCs were exposed to neural induction medium, they rapidly reshaped from a flat to a spherical morphology. Subsequently, we observed hBM-MSC-derived intermediate cells in which nuclear movements generate only one cell protrusion (white arrow) and hBM-MSC-derived intermediate cells in which nuclear movements alternately generate one or two cellular protrusions (yellow arrows).

**Additional file 5: Movie S3.** When an hBM-MSC-derived intermediate cell has a nucleus without lobes, its movement within the cell generates only one cell protrusion. Related to Fig. 1B. Time-lapse phase-contrast and fluorescence microscopy imaging revealed that when an hBM-MSC-derived intermediate cell has a nucleus without lobes, its movement within the cell generates only one cell protrusion.

**Additional file 6: Figure S3.** Changes in nuclear positioning are not attributable to the cell body undergoing rotation or attempted migration. Time-lapse imaging revealed that non-migratory cells in which tail-less nuclei (**A**), tailed nuclei (**B**) and lobed nuclei (**C**) can change positions, while cell body projections (arrows) remain in the same cell position. Scale bar: 10 μm. The number at the top indicates the time since the time-lapse image began. Elapsed time is displayed in the format (hours:minutes).

**Additional file 7: Movie S4.** Changes in the positioning of tail-less nuclei are not attributable to the cell body undergoing rotation or attempted migration. Related to Fig. S3A. Time-lapse imaging revealed that tail-less nuclei can change positions, while cell body projections (arrows) remain in the same cell positions.

**Additional file 8: Movie S5.** Changes in the positioning of tailed nuclei are not attributable to the cell body undergoing rotation or attempted migration. Related to Fig. S3B. Time-lapse imaging revealed that tailed nuclei can change positions, while cell body projections (arrows) remain in the same the cell positions.

**Additional file 9: Movie S6.** Changes in the positioning of lobed nuclei are not attributable to the cell body undergoing rotation or attempted migration. Related to Fig. S3C. Time-lapse imaging revealed that lobed nuclei can change positions, while cell body projections (arrows) remain in the same cell positions.

**Additional file 10: Movie S7.** Tail-less nuclei formation (PhC/H2B-eGFP). Related to Fig. 4. Time-lapse phase-contrast and fluorescence microscopy imaging showed that tail-less nuclei are formed by a single nuclear remodeling sequence that occurs as the cell reshapes from a flat to a spherical morphology, positioning the nucleus in a peripheral position within the cell. Subsequently, the tail-less nucleus began to move within the hBM-MSC-derived intermediate cells.

**Additional file 11: Movie S8.** Tail-less nuclei formation (H2B-eGFP). Related to Fig. 4. Time-lapse fluorescence microscopy imaging revealed that tail-less nuclei are formed by a single nuclear remodeling sequence that occurs as the cell reshapes from a flat to a spherical morphology.

**Additional file 12: Movie S9.** Tailed nuclei formation by one nuclear remodeling sequence. Time-lapse fluorescence microscopy imaging showed that tail-less nuclei can be formed by a single nuclear remodeling sequence.

**Additional file 13: Movie S10.** Tailed nuclei formation by two nuclear remodeling sequences (PhC/H2B-eGFP). Related to Fig. 5. Time-lapse phase-contrast and fluorescence microscopy imaging revealed that first a nuclear remodeling sequence occurs as the cell reshapes from a flat to a spherical morphology, positioning the nucleus in a peripheral position within the cell. Next, the cell nucleus moves and undergoes a second nuclear sequence in which a tailed process in the nucleus is formed. Finally, the tailed nucleus began to move within the hBM-MSC-derived intermediate cells.

**Additional file 14: Movie S11.** Tailed nuclei formation by two nuclear remodeling sequences (H2B-eGFP). Related to Fig. 5. Time-lapse fluorescence microscopy imaging showed that first, a nuclear remodeling sequence occurs as the cell reshapes from a flat to a spherical morphology. Next, the cell nucleus moves and undergoes a second nuclear sequence in which a tailed process in the nucleus is formed.

**Additional file 15: Movie S12.** Lobed nuclei formation by one nuclear remodeling sequence. Related to Fig. S4A. Time-lapse fluorescence microscopy imaging revealed that lobed nuclei can be formed by a single nuclear remodeling sequence.

**Additional file 16: Movie S13.** Lobed nuclei formation by two nuclear remodeling sequences (PhC/H2B-eGFP). Related to Fig. 6. Time-lapse phase-contrast and fluorescence microscopy imaging showed that first, a nuclear remodeling sequence occurs as the cell reshapes from a flat to a spherical morphology, positioning the nucleus in a peripheral position within the cell. Subsequently, the cell nucleus moves and undergoes a second nuclear sequence in which a lobed nucleus is formed. Afterwards, the lobed nuclei began to move within the hBM-MSC-derived intermediate cells. Finally, we also noted that lobed nuclei can switch shape while moving within the cell, becoming tailed nuclei.

**Additional file 17: Movie S14.** Lobed nuclei formation by two nuclear remodeling sequences (H2B-eGFP). Related to Fig. 6. Time-lapse fluorescence microscopy imaging revealed that a nuclear remodeling sequence first occurs. Subsequently, the cell nucleus moves and undergoes a second nuclear sequence in which a lobed nucleus is formed. Afterwards, the lobed nuclei began to move within the hBM-MSC-derived intermediate cells. Finally, we also noted that lobed nuclei can switch shape while moving within the cell, becoming tailed nuclei.

**Additional file 18: Movie S15.** hBM-MSC-derived intermediate cells interact with each other through their cell nuclei. Related to Fig. 7. Time-lapse phase-contrast and fluorescence microscopy imaging showed that hBM-MSC-derived intermediate cell nuclei move within the cell generating cellular protrusions as they attempt to contact the cells around them, mainly observing interactions between the nuclei.

**Additional file 19: Figure S4.** The tails of the tailed nuclei can move within the hBM-MSC-derived intermediate cells, switching shape and size and even move in different z-planes. **A** Time-lapse imaging revealed that as the tailed nuclei move within the cell, the tails can switch shape and size (arrows). **B** Time-lapse images also showed that the tails even appear to move in different *z*-planes. Scale bar: 10 μm. The number at the top indicates the time since the time-lapse image began. Elapsed time is displayed in the format (hours:minutes).

## Data Availability

All data generated or analysed during this study are included in this published article [and its Additional files].
