# Supplementary figures and images for: The nuclei of human adult stem cells can move within the cell and generate cellular protrusions to contact other cells
Source: Stem Cell Res Ther. 2024 Feb 7;15:32. doi: 10.1186/s13287-024-03638-y (PMC10848534; doi:10.1186/s13287-024-03638-y)

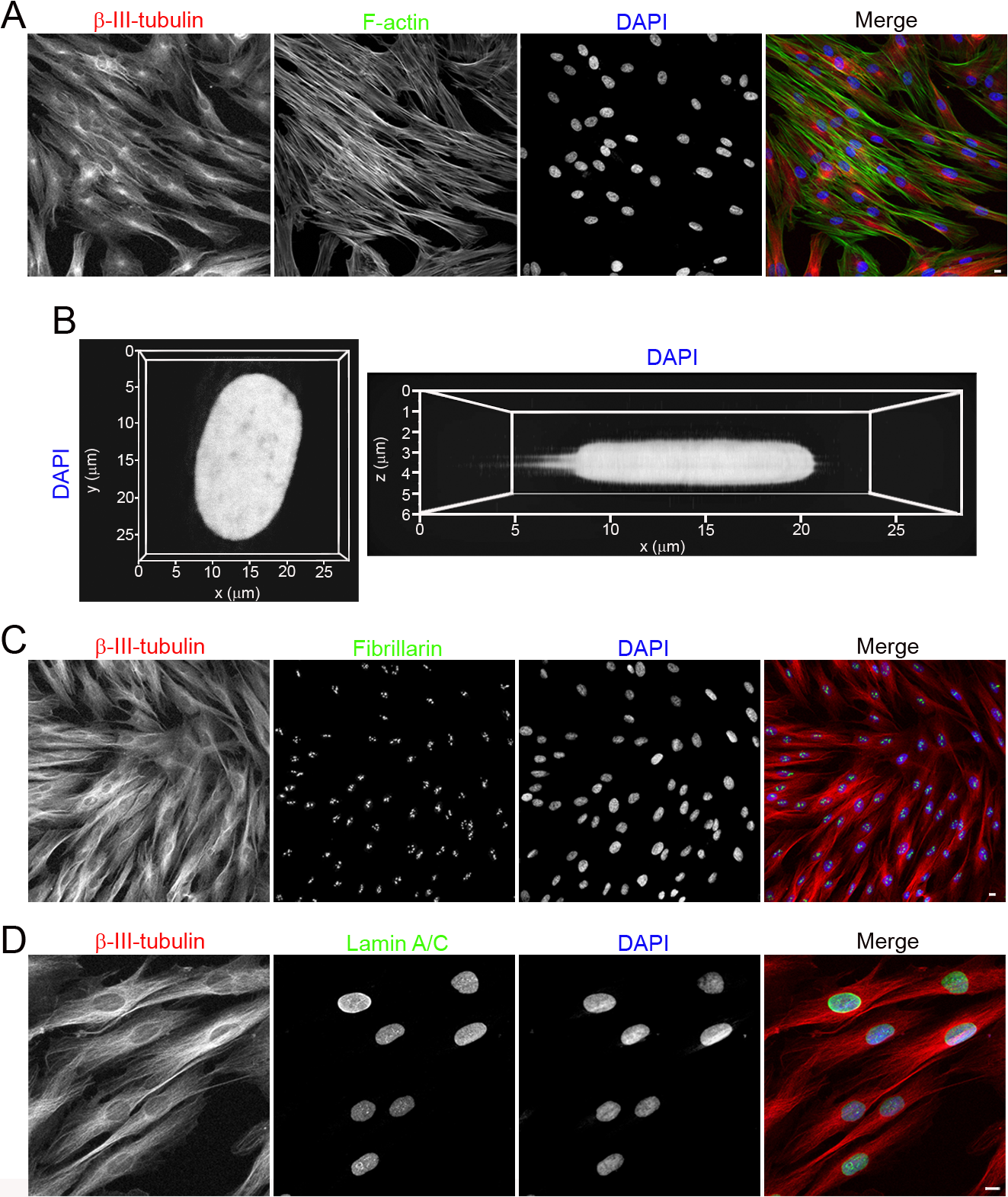

Supplement: Supplementary file 1 — Additional file 1: Figure S1. Morphology of hBM-MSCs cultured in basal medium. A Undifferentiated hBM-MSCs exhibited a fibroblast-like morphology with β-III tubulin microtubules and actin microfilaments oriented parallel to the longitudinal axis of the cell. B During interphase, hBM‑MSCs displayed a flattened, ellipsoidal nucleus, often located in the center of the cell. C Distribution of fibrillarin-positive specks in the nuclei of undifferentiated hBM-MSCs. D Immunocytochemical analysis revealed that the inner surface of the nuclear envelope is lined by the nuclear lamina. Scale bar: 10 μm. [file 13287_2024_3638_MOESM1_ESM.tif]

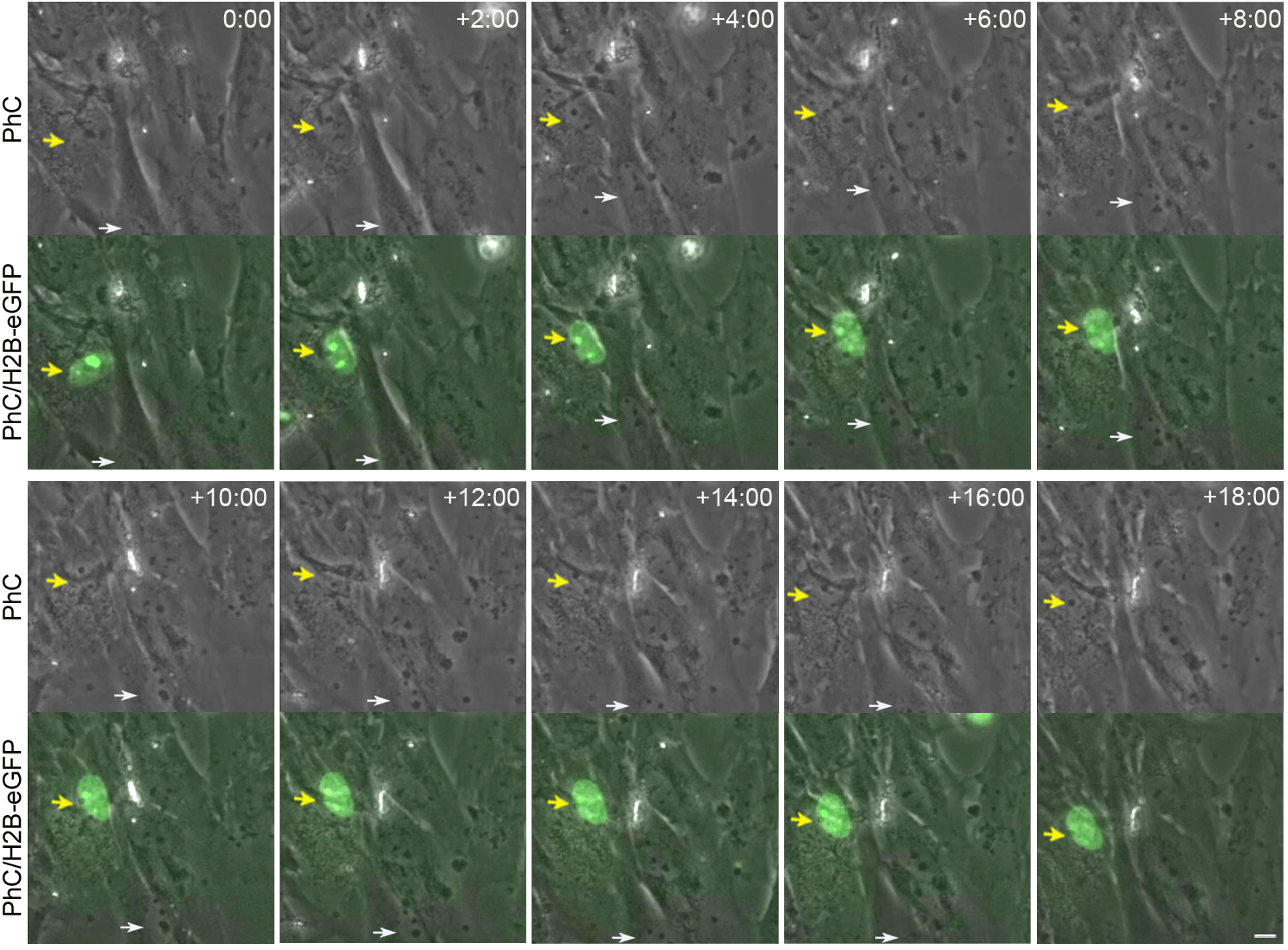

Supplement: Supplementary file 2 — Additional file 2: Figure S2. Spontaneous neural-like differentiation was not detected in H2B-GFP-transfected hBM-MSCs without neuronal induction. Time-lapse imaging revealed that H2B-GFP-transfected hBM-MSCs do not spontaneously differentiate into neural-like cells when cultured in a non-neuronal induction medium. The nuclei of the transfected (yellow arrows) and non-transfected cells (white arrows) did not change shape or generate cellular protrusions. Scale bar: 10 μm. The number at the top indicates the time since the time-lapse image began. Elapsed time is displayed in the format (hours:minutes). [file 13287_2024_3638_MOESM2_ESM.tif]

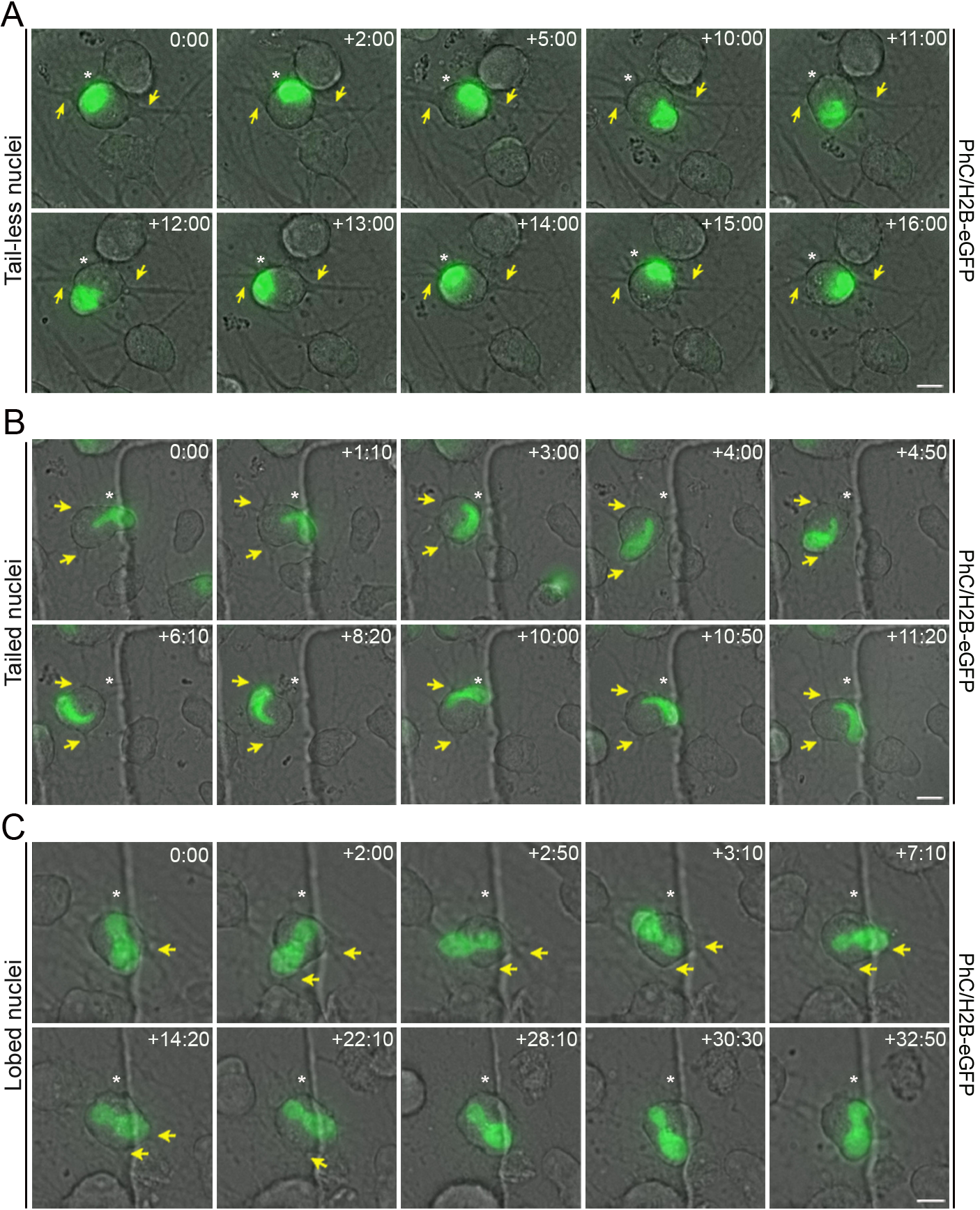

Supplement: Supplementary file 6 — Additional file 6: Figure S3. Changes in nuclear positioning are not attributable to the cell body undergoing rotation or attempted migration. Time-lapse imaging revealed that non-migratory cells in which tail-less nuclei (A), tailed nuclei (B) and lobed nuclei (C) can change positions, while cell body projections (arrows) remain in the same cell position. Scale bar: 10 μm. The number at the top indicates the time since the time-lapse image began. Elapsed time is displayed in the format (hours:minutes). [file 13287_2024_3638_MOESM6_ESM.tif]

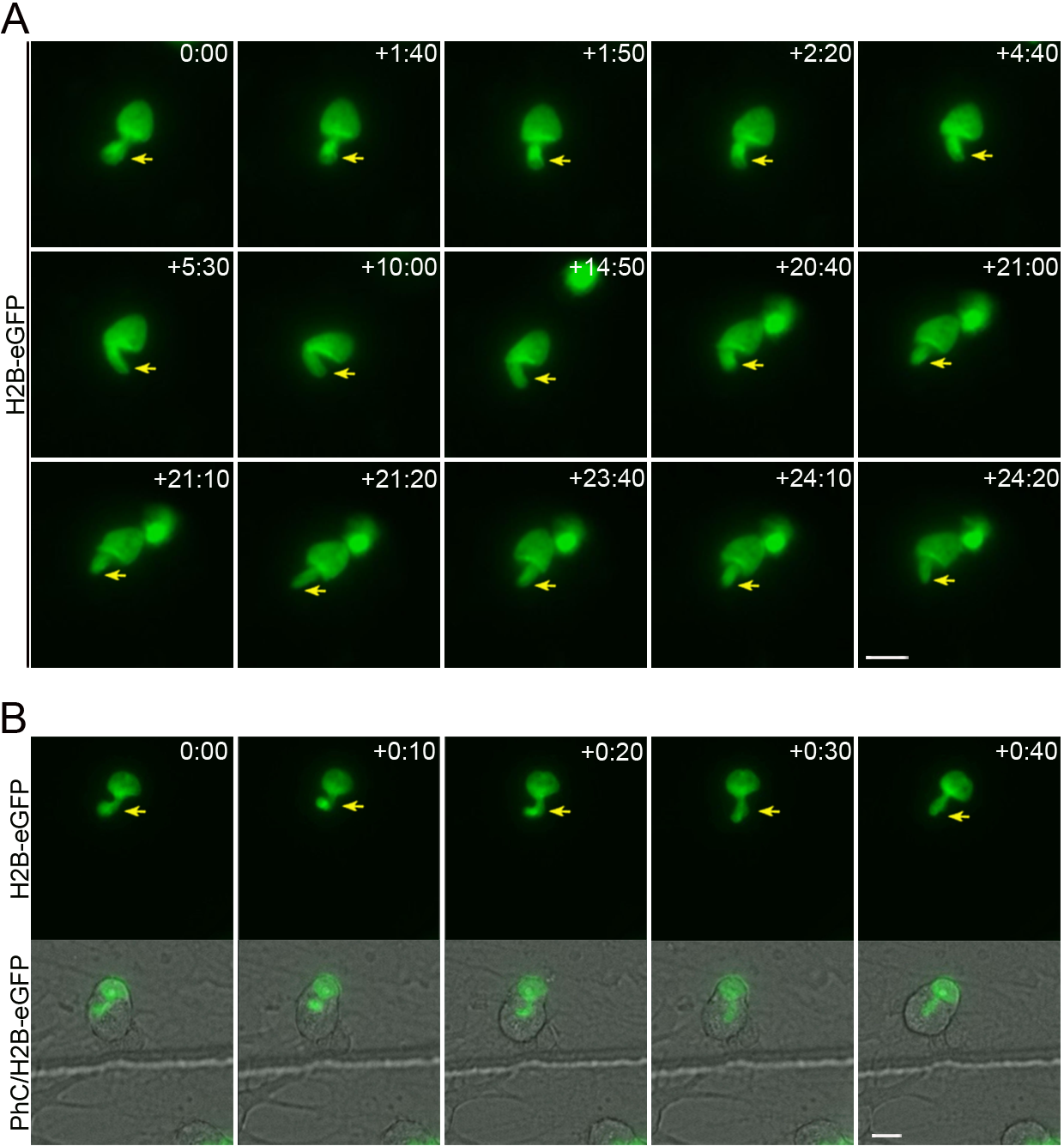

Supplement: Supplementary file 19 — Additional file 19: Figure S4. The tails of the tailed nuclei can move within the hBM-MSC-derived intermediate cells, switching shape and size and even move in different z-planes. A Time-lapse imaging revealed that as the tailed nuclei move within the cell, the tails can switch shape and size (arrows). B Time-lapse images also showed that the tails even appear to move in different z-planes. Scale bar: 10 μm. The number at the top indicates the time since the time-lapse image began. Elapsed time is displayed in the format (hours:minutes). [file 13287_2024_3638_MOESM19_ESM.tif]
